# The role of place of residency in childhood immunisation coverage in Nigeria: analysis of data from three DHS rounds 2003–2013

**DOI:** 10.1186/s12889-020-8170-6

**Published:** 2020-01-29

**Authors:** Olayinka Aderopo Obanewa, Marie Louise Newell

**Affiliations:** 1 Global Health Research Institute, School of Human Development and Health, Faculty of Medicine, University of Southampton, Southampton, SO17 1BJ UK; 2 School of Public Health, Faculty of Health Sciences, University of Witwatersrand, Johannesburg, South Africa

## Abstract

**Background:**

In 2017, about 20% of the world’s children under 1 year of age with incomplete DPT vaccination lived in Nigeria. Fully-immunised child coverage (FIC), which is the percentage of children aged 12–23 months who received all doses of routine infant vaccines in their first year of life in Nigeria is low. We explored the associations between child, household, community and health system level factors and FIC, in particular focussing on urban formal and slum, and rural residence, using representative Nigeria Demographic Health Survey (NDHS) data from 2003, 2008 and 2013.

**Method:**

Multilevel logistic regression models were applied for quantitative analyses of NDHS 2003, 2008 and 2013 data, singly, pooled overall and stratified by rural/urban, and within urban by formal and slum. We also quantify Population Attributable Risk (PAR) of FIC.

**Results:**

FIC for rural, urban formal and slum rose from 7.4, 25.6 and 24.9% respectively in 2003 to 15.8, 45.5 and 38.5% in 2013, and varied across sociodemographics. In pooled NDHS analysis, overall and stratified, final FIC adjusted odds (aOR) were: 1. Total population - delivery place (health facility vs home, aOR = 1.13, 95% CI = 0.73–1.73), maternal education (higher vs no education, aOR = 3.92, 95% CI = 1.79–8.59) and place of residence (urban vs rural, aOR = 1.69, 95% CI = 0.89–3.22). 2. Rural, urban formal and slum stratified: **A**.Rural – delivery place (aOR = 1.47, 95% CI = 1.12–1.94), maternal education (aOR = 4.99, 95% CI = 2.48–10.06). **B**.Urban formal - delivery place (aOR = 2.62, 95% CI = 1.43–4.79), maternal education level (aOR = 9.18, 95% CI = 3.05–27.64). **C**.Slums - delivery place (aOR = 5.39, 95% CI = 2.18–13.33), maternal education (aOR = 5.03, 95% CI = 1.52–16.65). The PAR revealed the highest percentage point increase in FIC would be achieved in all places of residence by maternal higher education: rural-38.15, urban formal-22.88 and slum 23.76, while non-attendance of antenatal care was estimated to lead to the largest reduction in FIC.

**Conclusion:**

Although low FIC in rural areas may be largely due to lack of health facilities and immunisation education, the intra-urban disparity is mostly unexplained, and requires further qualitative and interventional research. We show the FIC point increase that can be achieved if specific sociodemographic variable (risk) are addressed in the various communities, thus informing prioritisation of interventions.

## Background

Childhood immunisation is a cost-effective public health tool [1], resulting in the eradication of small pox, and elimination of polio in many countries, and drastically reducing morbidity and mortality caused by Diphtheria, Poliomyelitis, Pertussis, Tetanus, Measles and, more recently, *Haemophilus influenzae* type b, *Streptococcus pneumoniae*, Rotavirus, and Hepatitis B [1, 2]. However, the burden of vaccine-preventable child deaths remains substantial [3].

Nigeria has Africa’s largest under age 1 year population, but one of the lowest immunisation coverage [4, 5]; about 20% of the world’s children under 1 year of age with incomplete DPT vaccination in 2017 lived in Nigeria [4]. Maternal education, antenatal care attendance and place of delivery, religion and place of residence have been reported to be associated with childhood vaccination in Nigeria [6–9].

Immunisation rates are generally worse in rural than urban areas [6–9], and studies in Asia and the near East [10], India [11], Kenya [12] and Nigeria [13] have reported lower rates of immunisation coverage in slums than in formal urban areas. The rapid urbanisation in Nigeria may become a further barrier to childhood immunisation uptake, with growth of urban slums [14, 15], overstretched urban health facilities [16], leading to decreased health service quality and reduced access to health and immunisation services [13]. Hence the need to seek evidence to first, maintain and improve the relatively higher urban immunisation coverage compared to rural coverages, increase the rural coverages and lastly, reduce the prevalence of urban formal and slum immunisation disparity in Nigeria.

We will contribute to understanding of the associations between child, mother/family, community and health system level factors and immunisation coverage of routinely-provided immunisation in infancy, in particular focussing on urban formal and slum, and rural place of residence, in Nigeria, with the use of multilevel logistic regression and population attributable risk analyses on the available Nigeria Demographic Health Survey (NDHS) data from 2003, 2008 and 2013.

## Method

### Datasets

Nationally representative Demographic and Health Surveys (DHS) were carried out in Nigeria in 2003, 2008 and 2013. DHS population samples were randomly selected in a three-stage probability sampling process, with targeted number of households based on Nigeria’s population census [17–19]. These DHS reports are publicly available; datasets are accessible upon application including study aim and analytical plans from DHS MEASURE (https://www.dhsprogram.com/).

The outcome of interest here was fully immunised child coverage (FIC), a dichotomous variable defined as receipt of all routine infant vaccines (Bacillus Calmette Guerin (one dose), Oral Polio Vaccine (three doses), Diphtheria-Pertussis-Tetanus (three doses), Measles (one dose)) and reported for children aged 12–23 months [17–19]. This information was determined from the vaccination cards, and where not available from mothers’ recall. Sociodemographic variables were selected based on the reviewed literature and authors’ experience, and grouped into three levels: child, household and community; variables were either taken straight from the DHS or recoded as appropriate:

#### Child level

birth order (first, second and third, fourth and fifth, sixth or more), sex (female, male), place of delivery (health facility, and home/other), maternal antenatal care attendance (at least one ANC visit, none, don’t know/Missing).

#### Household factors

maternal education (no formal education/primary/secondary/higher), sex of household head (female/male), religion (Christianity/Islam/traditionalist and others) as provided in DHS. Maternal employment status (no/yes), maternal age at the child’s birth (14–19/20–29/30–39/40–49 year), maternal marital status (never married/married/no longer married), who decides on expenditure of maternal income (mother alone/mother and spouse/spouse alone/no income mother), media exposure (no/yes) and urban/rural-specific Household wealth (poor/middle/rich) were recoded from the raw DHS data.

#### Community level factors

place of residence (rural, urban formal, urban slum): two (improved water and sanitation) of the five UN HABITAT slum indicators (durable housing, overcrowding and security of tenure) [20, 21] were used to stratify the urban settlement. The absence of information on the house wall type in the 2003 DHS led to non-inclusion of the wall as an indicator. The roof or floor quality was not chosen because as people become richer, their roofs and floors may be changed irrespective of where they live and this indicator was thus considered less appropriate in this setting. With the culture of “co-sleeping” with children and polygamous families being common in all settings, overcrowding was deemed not appropriate to define a slum household. Finally, information on the indicator of security of tenure was not available in the DHS datasets. Regions: Northcentral, Northeast, Northwest, Southeast, Southsouth and Southwest. Distance to the nearest health facility was grouped as reported a big problem or no/not a big problem. Interaction terms were developed with place of residence as appropriate.

### Statistical methods

The three DHS datasets were used in two ways: 1. Separate and pooled analyses of the three NDHS datasets to identify the patterns of association between the FIC and sociodemographic factors, quantifying the independent association with place of residence, and differences over time; 2.Stratified analysis (rural, urban formal and urban slums) of the pooled dataset.

Descriptive analysis was expressed as numbers, frequency and percentages; differences in immunisation coverage between categories of the sociodemographic variable were tested using the chi-square test.

The association between variables and the dependent variable, FIC, was assessed in logistic regression. Variables not statistically significantly (*p*-value > 0.05) associated with FIC in univariate analysis, or which displayed multicollinearity using the variance inflation factor (VIF) were not included in the regression models. In the pooled datasets for the first set of analyses, multilevel logistic regression included four models: Model 0 had place of residence and DHS year, Model 1 included child level factors, Model 2 child and household factors and the full Model 3 child, household, community factors and interaction terms for place of residence with maternal education level, household wealth, maternal ANC attendance, place of child delivery and mothers birth age. These five interaction terms were based on their significant association with full immunisation. In the stratified analyses, the three-level multilevel logistic regression included four models: Model 0 had only the dependent variable (FIC), Model 1 included child level factors, Model 2 child and household factors and the full Model 3 child, household and community factors. Model goodness of fit was assessed with the Akaike Information Criterion (AIC) [22, 23].

Finally, we estimated the population attributable risk (PAR) [22, 23] to explore the increase or decrease in FIC that can be achieved with optimum levels of specific sociodemographic variables (eg all mothers having higher education, all deliveries being in the health facilities) in any place of residence. PAR was calculated after fitting a simple logistic regression of FIC and each sociodemographic variable. The PAR values are presented in percentages and 95% significant confidence interval for each sociodemographic variable category in the different places of residence. All statistical analyses were done with STATA version 14.

## Results

### Description

The number of households interviewed rose from 7225 in 2003, to 34,070 in 2008, and 38,522 in 2013, with a total of 79,817 in the pooled dataset (Table 1). The number of children aged 12–23 months increased from 999 in 2003 to 5900 in 2013. In all three surveys, about two-thirds of the children resided in rural areas; 15.5% of all children aged 12–23 months in the pooled dataset resided in slum areas. FIC was lowest in rural areas, with slum FIC coverage higher than in rural but lower than in urban formal areas.

**Table 1:** Description of the 2003, 2008 and 2013 Nigeria Demographic and Health Surveys

	Demographic and Health Surveys			
	2003	2008	2013	Pooled
Number of clusters (communities)	365	888	904	2157
Number of households				
Selected	7.864	36,298	40,320	84,482
Occupied	7327	34,644	38,904	80,875
Interviews conducted	7225	34,070	38,522	79,817
Response rate (%)	98.6	98.3	99.0	98.7
Total number of children aged 12–23 months (% of total)				
Rural	687 (68.8)	3447 (69.7)	3787 (64.2)	7920 (66.9)
Formal urban	104 (10.4)	781 (15.8)	1203 (20.4)	2087 (17.6)
Slum	208 (20.8)	718 (14.5)	911 (15.4)	1836 (15.5)
Total	999 (100)	4945 (100)	5900 (100)	11,844 (100)
Fully Immunised Child coverage (%)				
Rural	7.4	16.2	15.8	15.2
Formal urban	25.6	42.7	45.5	43.5
Slum	24.9	31.9	38.5	34.4
Total	12.9	22.7	25.3	23.2

### Multilevel analysis of the pooled dataset

Place of residence was significantly associated with FIC, with urban children more likely to be fully immunised than children in rural communities, and those in urban formal areas having higher odds than those in slums (Table 2). However, with the introduction of community variables and interaction terms in Model 3, FIC for an urban child (adjusted Odds Ratio (aOR) =1.60, 95% CI = 0.60–4.24) compared to the rural child remained higher, but was no longer statistically significant. FIC odds significantly increased over time, Model 0, with 2013 NDHS (aOR = 3.08, 95% CI = 2.21–4.29) higher than 2008 NDHS (aOR = 2.01, 95% CI = 1.46–2.77), which was higher than the reference, 2003 NDHS; this pattern was also seen in the subsequent models.

**Table 2:** Association between socio-demographic factors and full immunisation status (assessed at 12–23 months) in Nigeria, multivariable logistic regression analysis (pooled DHS data)

Variable	Model 0		Model 1		Model 2		Model 3	
	Adjusted Odds ratio 95% Confidence interval	*P* value	Adjusted Odds ratio 95% Confidence interval	*P* value	Adjusted Odds ratio 95% Confidence interval	*P* value	Adjusted Odds ratio 95% Confidence interval	*P* value
Place of Residence								
Rural	1.00		1.00		1.00		1.00	
Formal	6.37 (4.63,8.78)	0.001	2.35 (1.85,2.97)	0.001	1.80 (1.41,2.28)	0.001	1.69 (0.89,3.22)	0.108
Slum	5.13 (3.81,6.91)	0.001	1.93 (1.54,2.42)	0.001	1.76 (1.38,2.25)	0.001	1.45 (0.44,4.78)	0.541
DHS								
2003	1.00		1.00		1.00		1.00	
2008	2.01 (1.46,2.77)	0.001	2.99 (2.14,4.17)	0.001	2.77 (1.84,4.16)	0.001	3.27 (2.12,5.03)	0.001
2013	3.08 (2.21,4.29)	0.001	4.12 (2.92,5.82)	0.001	3.84 (2.53,5.83)	0.001	4.51 (2.90,7.02)	0.001
CHILD LEVEL FACTORS								
Birth order								
> =6			1.00		1.00		1.00	
1			1.60 (1.27,2.01)	0.001	1.82 (1.30,2.55)	0.001	1.69 (1.19,2.40)	0.004
2–3			1.52 (1.23,1.87)	0.001	1.47(1.11,1.94)	0.007	1.39 (1.04,1.86)	0.028
4–5			1.24 (1.00,1.54)	0.053	1.19 (0.92,1.54)	0.177	1.14 (0.87,1.48)	0.342
Place of delivery								
Home			1.00		1.00		1.00	
Health facility			3.75 (3.00,4.69)	0.001	2.07 (1.69,2.54)	0.001	1.13 (0.73,1.73)	0.591
Antenatal attendance								
No			1.00		1.00		1.00	
Yes			11.37(8.09,15.99)	0.001	7.44 (5.31,10.42)	0.001	8.45 (5.21,13.69)	0.001
Don’t know			7.66(5.32,11.03)	0.001	4.81 (3.30,7.00)	0.001	6.45 (2.82,14.72)	0.001
HOUSEHOLD LEVEL FACTORS								
Maternal education level								
No education					1.00		1.00	
Primary					2.14 (1.66,2.76)	0.001	1.77 (1.29,2.44)	0.001
Secondary					3.43 (2.57,4.58)	0.001	2.67 (1.64,4.36)	0.001
Higher					5.08 (3.36,7.68)	0.001	3.92 (1.79,8.59)	0.001
Sex of Household head								
Male					1.00		1.00	
Female					1.16(0.91,1.48)	0.242	1.10(0.85,1.41)	0.502
Religion								
Islam					1.00		1.00	
Christian					3.22 (2.50,4.17)	0.001	2.36 (1.81,3.08)	0.001
Traditionalist/ others					0.84 (0.51,1.38)	0.489	0.84 (0.50,1.40)	0.499
Mother employment status								
No					1.00		1.00	
Yes					1.22 (0.93,1.60)	0.154	1.15 (0.87,1.52)	0.327
Maternal age at the child’s birth								
14–19					1.00		1.00	
20–29					2.22(1.62,3.04)	0.001	2.35 (1.61,3.45)	0.001
30–39					2.85(1.95,4.16)	0.001	3.33(1.84,6.02)	0.001
40–49					3.33(1.98,5.60)	0.001	4.41(1.86,10.44)	0.001
Current marital status								
Never married					1.00		1.00	
Married/partner					0.69(0.43,1.12)	0.136	0.77(0.47,1.27)	0.304
No longer together					0.77(0.40,1.48)	0.438	0.93(0.47,1.83)	0.826
Decision maker on spending of mothers income								
No income mother /missing					1.00		1.00	
Mother alone					1.12 (0.86,1.47)	0.394	1.17 (0.88,1.55)	0.279
mother& spouse					1.25 (0.92,1.70)	0.163	1.19 (0.86,1.63)	0.301
spouse alone					0.77 (0.53,1.12)	0.174	0.76(0.51,1.12)	0.162
Media exposure								
No					1.00		1.00	
Yes					1.89 (1.51,2.36)	0.001	1.91 (1.50,2.41)	0.001
Household wealth								
Poor					1.00		1.00	
Moderate					1.14 (0.96,1.35)	0.129	1.30 (0.91,1.85)	0.155
Rich					1.31 (0.93,1.85)	0.126	1.69 (0.86,3.32)	0.126
COMMUNITY LEVEL FACTORS								
Region								
Northcentral							1.00	
Northeast							0.56 (0.40,0.78)	0.001
Northwest							0.30 (0.21,0.44)	0.001
Southeast							1.10 (0.78,1.55)	0.589
Southsouth							1.54 (1.11,2.14)	0.010
Southwest							0.90 (0.65,1.24)	0.523
Distance to nearest Health Facility Big problem No/Not a big problem							1.00 1.69 (1.37,2.09)	0.001
Interactions with place of residence Maternal Education							1.03(0.91,1.17)	0.643
Household wealth							0.90 (0.74,1.09)	0.289
ANC attendance							0.89 (0.69,1.13)	0.331
Place of delivery							1.38 (1.08,1.76)	0.010
Mothers birth age							0.92 (0.79,1.06)	0.238

After controlling for child, household, community level variables and fitting interaction terms in model 3, odds and significance of the relationship between FIC and independent variables were reduced but with similar intra-variable trends. Maternal antenatal care attendance (aOR = 8.45, 95% CI = 5.21–13.69) increased FIC odds. Maternal education was also associated with FIC: compared to mothers without formal education, FIC odds of mothers with primary education was 77% higher (aOR = 1.77, 95% CI = 1.29–2.44), and for mothers with secondary and with higher education odds increased by 167% (aOR = 2.67, 95% CI = 1.64–4.36) and 292% (aOR = 3.92, 95% CI = 1.79–8.59) respectively. Children of Christian households (aOR = 2.36, 95% CI = 1.81–3.08) were more likely to be fully immunised than those from Muslim homes. FIC odds increased by at least 135% with maternal age 20 years and above compared to age 14–19 years. Children from media-exposed homes (aOR = 1.91, 95% CI = 1.50–2.41) were at higher odds of being fully immunised than peers from households that were not. Children whose mothers resided in Northeast (aOR = 0.56, 95% CI = 0.40–0.78) or Northwest (aOR = 0.30, 95% CI = 0.21–0.44), and those who saw distance to the health facility as a big problem were less likely to be fully immunised.

Of the interactions between place of residence and maternal education (aOR = 1.03, 95% CI = 0.91–1.17), household wealth (aOR = 0.90, 95% CI = 0.74–1.09), maternal antenatal care (aOR = 0.89, 95% CI = 0.69–1.13), place of delivery (aOR = 1.38, 95% CI = 1.08–1.76) and maternal age at birth (aOR = 0.92, 95% CI = 0.79–1.06), only the interaction between place of residence and place of delivery was significant.

### Multilevel analysis of pooled NDHS dataset stratified by place of residence

The associations between FIC and sociodemographic variables were further explored in multilevel logistic regression analyses of the pooled dataset stratified by rural, formal urban and slum. Only Model 3 (final) is presented in Table 3 (with details on all Models in Additional file 1: Tables SA, SB and SC).

**Table 3:** Association between socio-demographic factors and full immunisation status (assessed at 12–23 months) in Rural, Urban formal and slum residence of Nigeria, Multilevel logistic regression analysis (DHS 2003, 2008 and 2013 data)

Variable /Category	Model 3 of places of residence		
	Rural (*n* = 7920)	Urban formal (*n* = 2087)	Slum (*n* = 1836)
	Adjusted Odd ratio / (95% C.I)	Adjusted Odd ratio / (95% C.I)	Adjusted Odd ratio / (95% C.I)
CHILD			
Birth order			
> =6	1.00	1.00	1.00
1	1.30 (0.81,2.08)	2.43 (1.01,5.87)	5.39 (1.37,21.29)
2–3	1.15 (0.78,1.68)	1.50 (0.72,3.12)	3.00 (1.00,9.13)
4–5	0.94 (0.66,1.34)	1.38 (0.69,2.75)	2.05 (0.78,5.40)
Place of delivery			
Home	1.00	1.00	1.00
Health facility	1.47 (1.12,1.94)	2.62 (1.43,4.79)	5.39 (2.18,13.33)
Antenatal attendance			
No	1.00	1.00	1.00
Yes	8.37 (5.34,13.12)	6.82 (2.29,20.34)	8.07 (2.15,30.25)
Don’t know	5.07 (3.06,8.41)	2.89 (0.94,8.88)	5.19 (1.21,22.37)
HOUSEHOLD			
Maternal education level			
No education	1.00	1.00	1.00
Primary	1.67 (1.19,2.35)	1.77 (0.83,3.79)	2.48 (1.02,6.05)
Secondary	2.49 (1.68,3.69)	4.57 (1.94,10.79)	4.46 (1.68,11.82)
Higher	4.99 (2.48,10.06)	9.18 (3.05,27.64)	5.03 (1.52,16.65)
Sex of Household head			
Male	1.00	1.00	1.00
Female	1.09 (0.76,1.54)	1.04 (0.60,1.94)	1.55 (0.62,3.86)
Religion			
Islam	1.00	1.00	1.00
Christian	2.63 (1.79,3.86)	1.59 (0.89,2.86)	5.69 (2.09,15.45)
Traditionalist/ others	0.20 (0.09,0.45)	0.40 (0.10,1.18)	0.28 (0.09,0.88)
Mother employment status			
No	1.00	1.00	1.00
Yes	1.16 (0.81,1.67)	1.78 (0.80,3.98)	0.41 (0.13,1.30)
Maternal age at the child’s birth			
14–19	1.00	1.00	1.00
20–29	1.76 (1.17,2.66)	5.25 (1.86,14.85)	2.18 (0.70,6.84)
30–39	2.19 (1.32,3.63)	6.64 (2.03,21.75)	2.63 (0.66,10.53)
40–49	2.05 (1.02,4.13)	9.87 (2.01,48.59)	5.40 (0.80,36.48)
Current marital status			
Never married	1.00	1.00	1.00
Married/partner	0.80 (0.42,1.54)	1.48 (0.42,5.22)	0.19 (0.03,1.33)
No longer together	1.14 (0.47,2.78)	2.93 (0.52,16.60)	0.05 (0.03,0.82)
Decision maker on spending of mothers income			
No income mother /missing	1.00	1.00	1.00
Mother alone	1.05 (0.73,1.50)	1.04 (0.48,2.26)	4.52 (1.34,15.30)
mother& spouse	1.42 (0.94,2.15)	0.65 (0.27,1.56)	3.00 (0.82,10.97)
spouse alone	0.80 (0.48,1.36)	0.43 (0.15,1.20)	2.17 (0.51,9.15)
Media exposure			
No	1.00	1.00	1.00
Yes	1.91 (1.43,2.54)	1.35 (0.65,2.79)	2.49 (0.95,6.53)
Household wealth			
Poor	1.00	1.00	1.00
Moderate	1.16 (0.91,1.48)	0.80 (0.50,1.27)	0.89 (0.51,1.57)
Rich	1.41 (0.87,2.29)	0.70 (0.32,1.53)	1.83 (0.22,15.58)
COMMUNITY			
Region			
Northcentral	1.00	1.00	1.00
Northeast	0.61 (0.39,0.95)	0.56 (0.20,1.55)	0.21 (0.07,0.66)
Northwest	0.26 (0.16,0.44)	0.85 (0.33,2.14)	0.20 (0.06,0.67)
Southeast	1.15 (0.70,1.92)	3.01 (1.19,7.57)	0.37 (0.11,1.21)
Southsouth	1.73 (1.11,2.71)	5.61 (1.88,16.71)	0.41 (0.13,1.21)
Southwest	0.85 (0.51,1.43)	1.12 (0.54,2.33)	0.88 (0.34,2.31)
Distance to nearest Health Facility			
Big problem	1.00	1.00	1.00
No/Not a big problem	1.76 (1.35,2.29)	1.77 (0.99,3.17)	2.21 (0.95,5.13)
Household variance (S.E)	0.104(0.164)	0.003(0.141)	0.131 (0.277)
Household ICC	0.245	0.090	0.098
Community variance (S.E)	0.212(0.135)*	0.343 (.123)*	0.374 (0.139)
Community ICC	0.200	0.095	0.133
Goodness of fit- AIC	5457	2185	1941

*S.E* standard error; *AIC* Akaike information criterion; *ICC* Intra Class Correlation; *C.I* Confidence interval:*p- Value < 0.05

In each setting, factors significantly associated with FIC were place of delivery, maternal ANC attendance, maternal education level and region. FIC was significantly associated with birth order in urban formal and slums, religion in rural and slums, maternal age in rural and urban formal, media exposure and distance to the health facility in rural areas, and with current maternal marital status and decision-maker of maternal income in slums. FIC was not significantly associated in any setting with sex of the household head, mother employment status and household wealth.

The increased FIC odds for health facility delivery was higher among urban (urban formal and slums) than rural dwellers, which was also true for the birth order associations. In contrast, in rural areas, maternal ANC attendance appeared more important than in urban formal and slum areas.

FIC significantly increased with higher maternal education level in all areas, with the size of the association largest in urban areas. This was also seen for mothers’ age at birth of the child. Compared to a child of a Muslim mother, a Christian child had a significantly higher odds of being fully immunised, especially in the slums. The association between FIC and household media exposure was significant only in rural areas.

Almost half of all children resided in the Northeast and Northwest and these had the lowest odds of being fully immunised in all settings. Distance to the nearest health facility when seeking health care was significantly associated with FIC in rural areas only.

The odds of a child being fully immunised varied across households and communities in the rural, urban formal and slum areas, with household and community factors explaining 24.5 and 20.0% variation in rural, 9.0 and 9.5% in urban formal and 9.8 and 13.3% in the slums respectively.

### Population attributable risk of the Sociodemographic variables on FIC in rural, urban formal and slum populations of Nigeria

To further understand the barriers of childhood immunisation, population attributable risk (PAR) analysis was deployed to investigate how the levels of FIC are associated with sociodemographic variables in a specific place of residence. Table 4 shows PARs for FIC in each place of residence for selected socio-demographic variables (PARs of all sociodemographic variables are in Additional file 1: Table SD). Non-attendance at antenatal care by mothers in urban formal areas reduced the average urban formal FIC percentage by 32 points. A 38 point increase over the average rural FIC percentage could be obtained if all rural mothers had higher education. Overall, variables had similar effect in each of the areas, either increasing or decreasing FIC, but with differing magnitude. FIC could be increased in rural, urban formal and slum by 19, 11 and 14 points respectively if all deliveries were at health facilities. FIC would be increased by 11, 5 and 4 points in rural, urban formal and slum populations respectively if all mothers had attended at least one antenatal care session.

**Table 4:** Population attributable risk of the Sociodemographic variables on FIC in rural, urban formal and slum populations of Nigeria

Variables/Risk factors	Rural (*n* = 7920)	Urban formal (*n* = 2087)	Slum (*n* = 1836)
	PAR in % /(95% C.I)	PAR in % /(95% C.I)	PAR in % /(95% C.I)
CHILD			
Birth order			
1	3.00 (1.18, 4.83)	7.99 (3.79, 12.16)	10.51 (6.11, 14.86)
2–3	2.06 (0.81, 3.31)	0.43 (−2.41, 3.27)	4.97 (1.84, 8.08)
4–5	- 0.75 (− 2.15, 0.66)	- 0.03 (− 4.07, 4.01)	- 2.22 (− 5.84,1.41)
> =6	- 3.60 (−4.84, − 2.36)	−11.86 (−16.55, − 7.11)	−15.81 (− 19.49,-12.08)
Place of delivery			
Home	- 6.28 (− 6.83, − 5.72)	− 19.16(− 21.87,-16.41)	−19.13(− 21.43,-16.81)
Health facility	18.78 (17.12, 20.43)	10.52 (9.02, 12.02)	13.51 (11.87,15.13)
Antenatal attendance			
No	−13.24 (−14.03,-12.44)	− 31.70(− 35.74, − 27.54)	− 28.94(− 32.26,-25.54)
Yes	10.88 (10.07,11.70)	4.66 (3.66, 5.67)	4.27 (3.26, 5.28)
Don’t know	6.96 (3.91, 9.99)	0.76 (− 0.73, 5.77)	2.86 (−3.44, 9.14)
HOUSEHOLD			
Maternal education level			
No education	−10.64 (−11.34, − 9.93)	−27.50(−30.75,-24.18)	− 23.74(− 26.40,-21.04)
Primary	5.49 (3.82, 7.15)	−07.84 (− 12.14,-3.52)	− 3.54 (− 7.39, 0.32)
Secondary	20.54 (18.53, 22.54)	9.13 (6.71, 11.53)	12.28 (9.35,15.18)
Higher	38.15 (31.17, 44.72)	22.88 (17.68, 27.95)	23.76 (18.09,29.27)
Maternal age at the child’s birth			
14–19	−6.58 (− 8.11, −5.05)	−21.18(− 27.25,-14.94)	−11.52(−17.53,-5.42)
20–29	2.72 (0.50, 1.04)	0.97 (−0.95, 2.89)	1.52 (−0.46, 3.49)
30–39	3.80 (2.38, 5.23)	3.71 (0.36, 7.05)	0.93 (−2.33, 4.18)
40–49	−0.77 (−4.21, 2.68)	1.49 (− 9.4,12.39)	− 0.60 (− 9.90, 8.72)
Media exposure			
No	−8.25 (−9.11, − 7.39)	−19.97(− 24.99,-14.84)	−19.50(−23.54,-15.39)
Yes	6.30 (5.64, 6.96)	2.81 (2.10, 3.53)	3.56 (2.82, 4.31)
COMMUNITY			
Region			
Northcentral	5.83 (3.86, 7.81)	−2.81 (−8.63, 3.02)	10.19 (5.31, 15.02)
Northeast	−6.00 (−7.25, −4.74)	−23.25(−28.20,-18.18)	−17.89(−21.21,-14.54)
Northwest	−12.21 (−13.08,-11.35)	−22.08(−26.37,-17.70)	−20.59(−24.05,-17.09)
Southeast	23.22 (19.12, 27.23)	11.46 (7.02, 15.84)	11.67 (5.24, 18.00)
Southsouth	20.15 (17.41, 22.86)	19.69 (13.38, 25.85)	10.78 (4.62, 16.85)
Southwest	12.60 (8.95, 16.22)	6.37 (2.95, 9.78)	21.05 (16.21, 25.80)

However, maternal primary education would increase FIC by 6 points in rural areas but reduce FIC by 8 and 4 points in urban and slum populations respectively. The FIC of children with mothers aged 14–19 years was reduced by 7 to 21 points depending on place of residence. The effect of being exposed to media on increased FIC was felt more in the rural (6 points) and least in urban formal (3 points), while a 4 point percentage difference was achieved in the slums. Being resident in any of the 3 southern regions increased the FIC percentage, while in two (Northeast and Northwest) of the 3 Northern regions, the risk of reduced FIC was increased. Each percentage point represents tens of eligible children: 79 in rural (*n* = 7920), 21 in urban formal (*n* = 2087) and 18 in slum (1836).

## Discussion

Using three national Demographic and Health surveys, 2003, 2008 and 2013, we quantify the associations between FIC and child, household and community factors in Nigeria. Allowing for child, household and community level factors, in multilevel logistic analysis of a pooled data set covering the three DHS, the odds of being fully immunised in 2008 was triple, and in 2013 it was 4.5-times that in 2003. FIC differed substantially by place of residence, with children in urban formal and slum areas being 69 and 45% respectively, more likely to be immunised than those in rural areas. Overall, and in each of the three place of residence strata separately, we found place of delivery, antenatal care, maternal education, maternal age at child birth, religion, place of residence, media exposure and distance to the health facility to be significantly associated with FIC. The pattern of the adjusted odds in overall pooled and stratified logistic models were similar, although the magnitude of odds varied. The pooled analyses provided quantification of changes over time, as well as reliable quantification of the associations with sociodemographic variables allowing for year and setting. Then stratified analyses allowed quantification of the association, highlighting potential differences by setting in these associations. In line with this adjusted odds, the PAR analysis quantified what could be achieved if these variables are optimised and provided more explanation of the FIC variation like health system related factors being more influential in the rural area compared to urban.

Overall, our results are in line with those from previous studies [6–9], based on more robust methodology we provided more details such as the association in the different places of residence and the improvement that could be obtained by ensuring all mothers obtain the level of the socio-demographic variable associated with optimum FIC.

### Child level factors

Our finding that children of lower birth orders were more likely to be fully immunised than higher birth orders confirm evidence from studies in Nigeria and India [6, 24–27]. Competing demands for family resources and time with increasing number of children likely explains at least part of this association [28]. In our study FIC was higher among children delivered in a health facility than those delivered at home in all three settings, similar to earlier studies [6–9], which were criticised on methodological grounds for including the 1999 NDHS data in their analyses [29]. Health facility delivery would likely increase the uptake of birth dose vaccines, BCG, OPV and HBV, and the provision of immunisation health messages, including date and place for subsequent immunisation sessions [9, 27, 30, 31]. Further, non-delivery in a health facility may be suggestive of distrust and lack of confidence in modern medicine and its providers [32]. Similarly, children of mothers who attended antenatal care during their pregnancy independently had higher FIC coverage and significantly greater odds of being fully immunised than children whose mothers did not attend antenatal care. Adding to previously reported evidence [9, 26, 31], this study provided the strength and pattern of the significance of the association and residence disparity between antenatal care and fully immunised odds in Nigeria, with stratified multilevel analysis that showed the FIC adjusted odds of delivery in health facility was highest in the slums. PAR analysis estimated the percentage point increase in FIC that can be achieved if all mothers had attended antenatal care.

### Household level factors

Maternal education level was consistently associated with FIC: as the mothers’ level of education increased, the child’s fully immunised odds increased, in line with results of several studies from Nigeria and elsewhere [6, 7, 17–19, 24, 25, 28, 32–45]. We additionally explored and quantified the pattern and trend of the association between maternal education and FIC status over the years in the general population and in each of the three settings and found its influence positive. Further analysis with PAR estimated that if maternal education was improved the whole population would benefit but the rural population stand to gain more. Increased maternal education is likely associated with increased health seeking behaviour, improved understanding of immunisation messages, knowledge of available immunisation delivery sites and having more money to cover the transport cost to health facilities [27, 46, 47].

Mother’s religion was also associated with the likelihood of the child to be fully immunised, such that a Christian child was several times more likely to be fully immunised than a Muslim child, with at least 25 point difference in their FIC irrespective of place of residence. This finding corroborated results from previous research in Nigeria and other countries [25, 48, 49]. Renne explained that Muslim leaders in Nigeria felt the vaccines were contaminated with HIV and anti-fertility substances aimed at reducing the Muslim population [48], while Taylor identified political and socioeconomic factors as reasons for acceptance or refusal of polio vaccine administration [49]. New evidence provided by this study was the lack of significance of the relationship between religion and fully immunised child odds in the urban formal areas. As found in earlier studies, the association between the age of the mother and fully immunised child status was significant [25, 41, 50]. Reasons suggested include lack of child care experience by young mother, experience gained by older mothers on the effectiveness of vaccines, and the effect of treating child illness on family income [6, 9]. Our study population was larger than those in previous studies, and nationally representative, thus increasing reliability of our findings.

Households with regular exposure to media had children with higher FIC than households without regular media exposure. This relationship had been documented as a determinant of childhood immunisation in previous research based on secondary analysis of NDHS datasets [9, 25]. The media provides information on the benefits of immunisation, health activities and location of health facilities, as such can serve as a tool to improve childhood immunisation [47].

Contrary to the findings of previous studies in Nigeria [9, 25], we found that the association between household wealth and childhood immunisation was not statistically significant, which may be attributed to the recoding of the wealth variable in this study to make it urban or rural relative, lest all rural population were classed as being poor relative to urban populations. Despite the free provision of immunisation services in public owned health facilities, there are still indirect costs that can be barriers, such as transport cost to the health facility that is far from the mothers, and for the low income earner's inability to be excused from work as a result of the considerable time spent for journey to and time spent in the health facility [8]. Also, the lack of money has been reported to hinder appropriate health care seeking behaviour [9].

### Community level factors

Overall, the Northern regions (Northcentral, Northeast and Northwest) had lower FIC adjusted odds than the Southern (Southeast, Southsouth and Southwest) regions, also reported by others [17–19, 40, 43]. Reasons suggested include higher education level and higher household wealth in the South than in the North, northerners are mostly Muslims and the southerners predominantly Christian, and the recent Muslim insurgency in the Northeast [9, 25]. However, across the six regions in Nigeria, there was high variability in the likelihood of being fully immunised. The Northwest region which is the most populous had the lowest FIC coverage and least fully immunised child odds compared to the other regions, while the Southsouth region had the highest fully immunised children odds. As expected, children of mothers who felt the distance to the nearest health facility to seek health was a big problem had lower FIC coverage and lower fully immunised child odds than children with mothers who did not see the distance as a big problem. This finding is in line with the results of several studies in Nigeria [9, 25, 51].

Our novel contribution lies in the PAR estimates, which showed that the population FIC in each of the three areas of residence was dependent on key sociodemographic exposure. Simply, the PAR is the additional number of children that would have been vaccinated if the sociodemographic variable had been maximised. Though no particular PAR trend was found across the places of residence, variables such as maternal education and media exposure that are linked to building knowledge about immunisation had the greatest effects in the rural areas, next was the slums and least in the urban formal settings. The PAR estimated highest percentage point increase in FIC can be achieved in all places of residence by giving all mothers higher education: rural (38.15), urban formal (22.88) and slum (23.76). Also, with all households having regular media exposure, the PAR was rural (6.30), urban formal (2.81) and slum (3.56). Hence, PAR analysis makes planning more realistic since target setting can be more correctly done as the number of children to be reached to increase the FIC by a percent as well as the variable to minimise or maximise to achieve it in each residence is known.

#### Study limitations

The analyses are based on data from retrospective cross-sectional surveys; immunisation data was collected for children aged between 12 and 23 months from the child’s immunisation card, with the mother recalling the information if the card was not available. Maternal report may be subject to recall bias, especially as event could have occurred more than a year before the interview. Similarly, maternal education level has been reported to be associated with recall bias, with the mothers of more educated level being more likely to accurately recall the child’s immunisation history. Thus, adjusting for maternal education in the multilevel logistic models reduced the effect of maternal education on the recall bias [52]. The household wealth variable in NDHS is a proxy based on the presence or otherwise of a number of assets, rather than on direct measurement of household income [53]. However, contrary to earlier studies, we calculated household wealth relative to the area of residence rather than for the overall population, and our wealth variable thus indicates wealth relative to the wealth of others living in the same area. Some of the selected NDHS variables were not perfectly aligned to the research question, for example the variable, “distance to the nearest health facility when seeking health care”, does not specifically refer to child immunisation. As the NDHS data provides information only on urban and rural place of residence, we used UN HABITAT guidelines to recode the urban data into formal and slum households. This may have introduced selection bias, as the DHS sampling process was based on projections from general census held several years earlier, and as such may not be fully representative of the population when the DHS was conducted.

## Conclusion

Nigeria’s fully immunised child coverage of about 23% is unacceptably low; utilisation of immunisation services is associated with sociodemographic variables acting at child, household, community and health system levels. However, even the most privileged sociodemographic groups do not achieve the recommended FIC coverage target. With this very low FIC coverage, the prevalence of vaccine preventable diseases will continue, leading to high child mortality rates and increased health expenditure by parents.

The pattern and coverage of being fully immunised across the sociodemographic variables were similar in all areas of residence (rural, urban formal and slum). The FIC was highest in urban formal, with the slums values slightly lower and the rural figures much lower. Although the very low rural coverages may be largely due to lack of health facilities and immunisation education as indicated by PAR analysis, the intra-urban disparity is mostly unexplained, and requires further qualitative and interventional research. But our epidemiological analysis has made it easier by estimating the risk as such quantifying the FIC point increase that can be achieved if specific sociodemographic variable (risk) are addressed in the various communities, thereby making prioritisation of interventions much easier.

In the meantime, pending further evidence, suggestions for improvement are location specific: Immunisation session reminders in the slums, engagement of Moslem leaders in slum and rural areas, improved immunisation education in all residences and re-establish outreach services in rural communities.

## Supplementary information

**Additional file 1: Table SA.**. Association between socio-demographic factors and full immunisation status (assessed at 12–23 months) in Rural Nigeria, Multilevel logistic regression analysis (DHS 2003, 2008 and 2013 data. **Table SB.** Association between socio-demographic factors and full immunisation status (assessed at 12–23 months) in Nigerian Urban formal Households, Multilevel logistic regression analysis (DHS 2003, 2008 and 2013 data. **Table SC.** Association between socio-demographic factors and full immunisation status (assessed at 12–23 months) in Nigerian Urban Slum Households, Multilevel logistic regression analysis (DHS 2003, 2008 and 2013 data. **Table SD**. Multivariate population attributable risk for fully immunised child coverage by place of residence.

## Data Availability

The anonymised data of the Demographic and Health Surveys conducted in Nigeria in 2003, 2008 and 2013 are available at (www.measuredhs.com), after approval by Measure DHS, but the analyses are available from the corresponding author on reasonable request.

## Abbreviations

ANC: Antenatal Care
aOR: Adjusted Odds Ratio
DHS: Demographic and Health Survey
DPT: Diphtheria Pertussis Tetanus
FIC: Fully Immunised Child
NDHS: Nigeria Demographic and Health Survey
OR: Odds Ratio
PAR: Population Attributable Risk
