# Supplementary material for: The role of place of residency in childhood immunisation coverage in Nigeria: analysis of data from three DHS rounds 2003–2013
Source: BMC Public Health. 2020 Jan 29;20:123. doi: 10.1186/s12889-020-8170-6 (PMC6988360; doi:10.1186/s12889-020-8170-6)
Supplement: Supplementary file 1 — Additional file 1: Table SA.. Association between socio-demographic factors and full immunisation status (assessed at 12–23 months) in Rural Nigeria, Multilevel logistic regression analysis (DHS 2003, 2008 and 2013 data. Table SB. Association between socio-demographic factors and full immunisation status (assessed at 12–23 months) in Nigerian Urban formal Households, Multilevel logistic regression analysis (DHS 2003, 2008 and 2013 data. Table SC. Association between socio-demographic factors and full immunisation status (assessed at 12–23 months) in Nigerian Urban Slum Households, Multilevel logistic regression analysis (DHS 2003, 2008 and 2013 data. Table SD. Multivariate population attributable risk for fully immunised child coverage by place of residence. [file 12889_2020_8170_MOESM1_ESM.docx]

## Supplementary Material

### Rural Multilevel Analysis

Table A: Association between socio-demographic factors and full immunisation status (assessed at 12-23 months) in Rural Nigeria, Multilevel logistic regression analysis (DHS 2003, 2008 and 2013 data

| Variable /Category | RURAL | | |
| --- | --- | --- | --- |
|  | Model 1 | Model 2 | Model 3 |
|  | Adjusted Odd ratio / (95% C.I) | Adjusted Odd ratio / (95% C.I) | Adjusted Odd ratio / (95% C.I) |
| CHILD |  |  |  |
| Birth order  >=6  1  2-3  4-5 | 1.00  1.28 (0.95,1.73)  1.30 (0.99,1.70)  1.02 (0.77,1.35) | 1.00  1.37 (0.87,2.17)  1.21 (0.84,1.76)  0.97 (0.69,1.37) | 1.00  1.30 (0.81,2.08)  1.15 (0.78,1.68)  0.94 (0.66,1.34) |
| Place of delivery  Home  Health facility | 1.00  2.94 (2.24,3.85) | 1.00  1.67 (1.28,2.17) | 1,00  1.47 (1.12,1.94) |
| Antenatal attendance  No  Yes  Don’t know | 1.00  13.93 (8.99,21.58)  8.5 7(5.36,13.70) | 1.00  9.14 (5.88,14.22)  5.73 (3.49,9.42) | 1.00  8.37 (5.34,13.12)  5.07 (3.06,8.41) |
| HOUSEHOLD |  |  |  |
| Maternal education level  No education  Primary  Secondary  Higher |  | 1.00  2.00 (1.44,2.79)  3.04 (2.06,4.49)  5.72 (2.88,11.38) | 1.00  1.67 (1.19,2.35)  2.49 (1.68,3.69)  4.99 (2.48,10.06) |
| Sex of Household head  Male  Female |  | 1.00  1.17 (0.83,1.64) | 1.00  1.09 (0.76,1.54) |
| Religion  Islam  Christian  Traditionalist/ others |  | 1.00  3.93 (2.69,5.74)  0.21 (0.10,0.47) | 1.00  2.63 (1.79,3.86)  0.20 (0.09,0.45) |
| Mother employment status  No  Yes |  | 1.00  1.18 (0.83,1.67) | 1.00  1.16 (0.81,1.67) |
| Maternal age at the child’s birth  14-19  20-29  30-39  40-49 |  | 1.00  1.90 (1.27,2.83)  2.45 (1.50,4.02)  2.25 (1.14,4.45) | 1.00  1.76 (1.17,2.66)  2.19 (1.32,3.63)  2.05 (1.02,4.13) |
| Current marital status  Never married  Married/partner  No longer together |  | 1.00  0.70 (0.37,1.32)  0.97 (0.41,2.31) | 1.00  0.80 (0.42,1.54)  1.14 (0.47,2.78) |
| Decision maker on spending of mothers income  No income mother /missing Mother alone  mother& spouse  spouse alone |  | 1.00  1.00 (0.71,1.42)  1.51 (1.00,2.26)  0.79 (0.48,1.32) | 1.00  1.05 (0.73,1.50)  1.42 (0.94,2.15)  0.80 (0.48,1.36) |
| Media exposure  No  Yes |  | 1.00  1.92 (1.46,2.53) | 1.00  1.91 (1.43,2.54) |
| Household wealth  Poor  Moderate  Rich |  | 1.00  1.20 (0.95,1.52)  1.48 (0.93,2.37) | 1.00  1.16 (0.91,1.48)  1.41 (0.87,2.29) |
| COMMUNITY |  |  |  |
| Region  Northcentral  Northeast  Northwest  Southeast  Southsouth  Southwest |  |  | 1.00  0.61 (0.39,0.95)  0.26 (0.16,0.44)  1.15 (0.70,1.92)  1.73 (1.11,2.71)  0.85 (0.51,1.43) |
| Distance to nearest Health Facility  Big problem No/Not a big problem |  |  | 1.00  1.76 (1.35,2.29) |
|  |  |  |  |
| Household variance (S.E) | 0.155(0.179) | 0.098(0.181) | 0.104(0.164) |
| Household ICC | 0.305 | 0.215 | 0.245 |
| Community variance (S.E) | 1.291 (0.163)* | 0.490(0.211)* | 0.212(0.135)* |
| Community ICC | 0.273 | 0.200 | 0.200 |
| Goodness of fit- AIC | 5924 | 5517 | 5457 |
|  |  |  |  |

S.E-standard error: AIC=Akaike information criterion: ICC=Intra Class Correlation: C.I=Confidence interval:*p- Value <0.05

### Multilevel analysis in Urban formal

Table B: Association between socio-demographic factors and full immunisation status (assessed at 12-23 months) in Nigerian Urban formal Households, Multilevel logistic regression analysis (DHS 2003, 2008 and 2013 data

| Variable /Category | Urban Formal | | |
| --- | --- | --- | --- |
|  | Model 1 | Model 2 | Model 3 |
|  | Adjusted Odd ratio / (95% C.I) | Adjusted Odd ratio / (95% C.I) | Adjusted Odd ratio / (95% C.I) |
| CHILD |  |  |  |
| Birth order  >=6  1  2-3  4-5 | 1.00  1.91 (0.98,3.74)  1.51 (0.83,2.77)  1.46 (0.77,2.77) | 1.00  2.14 (0.94,4.89)  1.35 (0.68,2.69)  1.31 (0.69,2.53) | 1.00  2.43 (1.01,5.87)  1.50 (0.72,3.12)  1.38 (0.69,2.75) |
| Place of delivery  Home  Health facility | 1.00  5.79 (2.73,12.27) | 1.00  2.56 (1.44,4.55) | 1,00  2.62 (1.43,4.79) |
| Antenatalattendance  No  Yes  Don’t know | 1.00  11.23 (3.59,35.17)  5.98 (1.91,18.71) | 1.00  6.07 (2.17,17.00)  3.02 (1.04,8.81) | 1.00  6.82 (2.29,20.34)  2.89 (0.94,8.88) |
| HOUSEHOLD |  |  |  |
| Maternal education level  No education  Primary  Secondary  Higher |  | 1.00  2.13(1.03,4.04)  5.43(2.33,12.65)  9.59(3.27,28.11) | 1.00  1.77 (0.83,3.79)  4.57 (1.94,10.79)  9.18 (3.05,27.64) |
| Sex of Household head  Male  Female |  | 1.00  1.16 (0.66,2.04) | 1.00  1.04 (0.60,1.94) |
| Religion  Islam  Christian  Traditionalist/ others |  | 1.00  2.68 (1.51,4.76)  0.51 (0.19,1.41) | 1.00  1.59 (0.89,2.86)  0.40 (0.10,1.18) |
| Mother employment status  No  Yes |  | 1.00  2.06 (0.95,4.46) | 1.00  1.78 (0.80,3.98) |
| Maternal age at the child’s birth  14-19  20-29  30-39  40-49 |  | 1.00  5.12 (1.89,13.89)  6.49 (2.09,20.18)  8.71 (1.92,39.56) | 1.00  5.25 (1.86,14.85)  6.64 (2.03,21.75)  9.87 (2.01,48.59) |
| Current marital status  Never married  Married/partner  No longer together |  | 1.00  1.20 (0.36,4.01)  1.94 (0.38,9.89) | 1.00  1.48 (0.42,5.22)  2.93 (0.52,16.60) |
| Decision maker on spending of mothers income  No income mother /missing Mother alone  mother& spouse  spouse alone |  | 1.00  0.80(0.39,1.66)  0.58 (0.25,1.35)  0.43 (0.16,1.14) | 1.00  1.04 (0.48,2.26)  0.65 (0.27,1.56)  0.43 (0.15,1.20) |
| Media exposure  No  Yes |  | 1.00  1.38 (0.69,2.75) | 1.00  1.35 (0.65,2.79) |
| Household wealth  Poor  Moderate  Rich |  | 1.00  0.83 (0.53,1.28)  0.69 (0.33,1.44) | 1.00  0.80 (0.50,1.27)  0.70 (0.32,1.53) |
| COMMUNITY |  |  |  |
| Region  Northcentral  Northeast  Northwest  Southeast  Southsouth  Southwest |  |  | 1.00  0.56 (0.20,1.55)  0.85 (0.33,2.14)  3.01 (1.19,7.57)  5.61 (1.88,16.71)  1.12 (0.54,2.33) |
| Distance to nearest Health Facility  Big problem No/Not a big problem |  |  | 1.00  1.77 (0.99,3.17) |
|  |  |  |  |
| Household variance (S.E) | 0.001(0.001) | 0.002 (0.101) | 0.003(0.141) |
| Household ICC | 0.160 | 0.100 | 0.090 |
| Community variance (S.E) | 0.636 (0.153) | 0.383 (.127)* | 0.343 (.123)* |
| Community ICC | 0.163 | 0.104 | 0.095 |
| Goodness of fit- AIC | 2297 | 2201 | 2185 |
|  |  |  |  |

S.E-standard error: AIC=Akaike information criterion: ICC=Intra Class Correlation: C.I=Confidence interval:*p- Value <0.05

### Multilevel analysis in urban slums

Table C: Association between socio-demographic factors and full immunisation status (assessed at 12-23 months) in Nigerian Urban Slum Households, Multilevel logistic regression analysis (DHS 2003, 2008 and 2013 data

| Variable /Category | Slum | | |
| --- | --- | --- | --- |
|  | Model 1 | Model 2 | Model 3 |
|  | Adjusted Odd ratio / (95% C.I) | Adjusted Odd ratio / (95% C.I) | Adjusted Odd ratio / (95% C.I) |
| CHILD |  |  |  |
| Birth order  >=6  1  2-3  4-5 | 1.00  6.89 (2.32,20.49)  4.52 (1.76,11.55)  2.88 (1.19,7.00) | 1.00  8.60 (2.03,36.41)  4.49 (1.41,14.29)  2.58 (0.95,6.97) | 1.00  5.39 (1.37,21.29)  3.00 (1.00,9.13)  2.05 (0.78,5.40) |
| Place of delivery  Home  Health facility | 1.00  16.94 (5.26,54.59) | 1.00  6.35 (2.49,16.19) | 1.00  5.39 (2.18,13.33) |
| Antenatalattendance  No  Yes  Don’t know | 1.00  15.04 (3.93,54.59)  11.03 (2.61,46.55) | 1.00  9.45 (2.47,36.05)  5.37 (1.24,23.24) | 1.00  8.07 (2.15,30.25)  5.19 (1.21,22.37) |
| HOUSEHOLD |  |  |  |
| Maternal education level  No education  Primary  Secondary  Higher |  | 1.00  3.04 (1.24,7.47)  5.13 (1.91,13.81)  5.76 (1.70,19.58) | 1.00  2.48 (1.02,6.05)  4.46 (1.68,11.82)  5.03 (1.52,16.65) |
| Sex of Household head  Male  Female |  | 1.00  1.55 (0.62,3.87) | 1.00  1.55 (0.62,3.86) |
| Religion  Islam  Christian  Traditionalist/ others |  | 1.00  6.68 (2.54,17.57)  0.26 (0.08,0.84) | 1.00  5.69 (2.09,15.45)  0.28 (0.09,0.88) |
| Mother employment status  No  Yes |  | 1.00  0.46 (0.15,1.45) | 1.00  0.41 (0.13,1.30) |
| Maternal age at the child’s birth  14-19  20-29  30-39  40-49 |  | 1.00  2.66 (0.83,8.51)  3.92 (0.94,16.31)  9.83 (1.37,70.62) | 1.00  2.18 (0.70,6.84)  2.63 (0.66,10.53)  5.40 (0.80,36.48) |
| Current marital status  Never married  Married/partner  No longer together |  | 1.00  0.15 (0.02,1.12)  0.03 (0.01,0.06) | 1.00  0.19 (0.03,1.33)  0.05 (0.03,0.82) |
| Decision maker on spending of mothers income  No income mother /missing Mother alone  mother& spouse  spouse alone |  | 1.00  4.91 (1.44,16.82)  3.53 (0.95,13.20)  2.27 (0.54,9.65) | 1.00  4.52 (1.34,15.30)  3.00 (0.82,10.97)  2.17 (0.51,9.15) |
| Media exposure  No  Yes |  | 1.00  2.79 (1.05,7.39) | 1.00  2.49 (0.95,6.53) |
| Household wealth  Poor  Moderate  Rich |  | 1.00  0.92 (0.52,1.61)  2.24 (0.27,18.45) | 1.00  0.89 (0.51,1.57)  1.83 (0.22,15.58) |
| COMMUNITY |  |  |  |
| Region  Northcentral  Northeast  Northwest  Southeast  Southsouth  Southwest |  |  | 1.00  0.21 (0.07,0.66)  0.20 (0.06,0.67)  0.37 (0.11,1.21)  0.41 (0.13,1.21)  0.88 (0.34,2.31) |
| Distance to nearest Health Facility  Big problem No/Not a big problem |  |  | 1.00  2.21 (0.95,5.13) |
|  |  |  |  |
| Household variance (S.E) | 0.08 (0.095) | 0.78 (0.264) | 0.131 (0.277) |
| Household ICC | 0.181 | 0.113 | 0.098 |
| Community variance (S.E) | 0.762 (.181)* | 0.427 (0.142) * | 0.374 (0.139) |
| Community ICC | 0.188 | 0.133 | 0.133 |
| Goodness of fit- AIC | 2051 | 1949 | 1941 |
|  |  |  |  |

S.E-standard error: AIC=Akaike information criterion: ICC=Intra Class Correlation: C.I=Confidence interval:*p- Value <0.05

Table D : Multivariate population attributable risk for fully immunised child coverage by place of residence

| Variables/Risk factors | Rural | Urban formal | Slum |
| --- | --- | --- | --- |
|  | PAR in % /(95% C.I) | PAR in % /(95% C.I) | PAR in % /(95% C.I) |
| CHILD |  |  |  |
| Birth order  1  2-3  4-5  >=6 | 3.00 (1.18, 4.83)  2.06 (0.81, 3.31)  -0.75 (-2.15, 0.66) -3.60 (-4.84, -2.36) | 7.99 (3.79, 12.16)  0.43 (-2.41, 3.27)  -0.03 (-4.07, 4.01)  -11.86 (-16.55, -7.11) | 10.51 (6.11, 14.86)  4.97 (1.84, 8.08)  -2.22 (-5.84,1.41)  -15.81 (-19.49,-12.08) |
| Place of delivery  Home  Health facility | -6.28 (-6.83, -5.72)  18.78 (17.12, 20.43) | -19.16(-21.87,-16.41)  10.52 (9.02, 12.02) | -19.13(-21.43,-16.81)  13.51 (11.87,15.13) |
| Antenatal attendance  No  Yes  Don’t know | -13.24 (-14.03,-12.44)  10.88 (10.07,11.70)  6.96 (3.91, 9.99) | -31.70(-35.74, -27.54)  4.66 (3.66, 5.67)  0.76 (-0.73, 5.77) | -28.94(-32.26,-25.54)  4.27 (3.26, 5.28)  2.86 (-3.44, 9.14) |
| HOUSEHOLD |  |  |  |
| Maternal education level  No education  Primary  Secondary  Higher | -10.64 (-11.34, -9.93)  5.49 (3.82, 7.15)  20.54 (18.53, 22.54)  38.15 (31.17, 44.72) | -27.50(-30.75,-24.18)  -07.84 (-12.14,-3.52)  9.13 (6.71, 11.53)  22.88 (17.68, 27.95) | -23.74(-26.40,-21.04)  -3.54 (-7.39, 0.32)  12.28 (9.35,15.18)  23.76 (18.09,29.27) |
| Sex of Household head  Male  Female | -1.21 (-1.52, -0.90)  11.89 (8.86,14.90) | -0.97(-1.86, -0.08)  0.62 (0.49, 11.94) | -1.33 (-2.08, -0.57)  12.07 (5.18, 18.84) |
| Religion  Islam  Christian  Traditionalist/ others | -9.10 (-9.80, -8.40)  16.60 (15.43, 17.75)  -13.71 (-15.27,-12.15) | -14.20(-16.83,-11.54)  12.23 (10.35, 14.10)  -23.58(-30.53,-16.39) | -12.42(-14.57 ,-10.26) 18.77 (16.29, 21.23)  -19.49(-24.31 ,-14.57) |
| Mother employment status  No  Yes | -4.51 (-5.60, -3.42)  2.17 (1.64, 2.69) | -8.04 (-11.63,-4.44)  2.92 (1.62, 4.22) | -8.07 (-11.28,-4.85)  3.37 (2.03, 4.72) |
| Maternal age at the child’s birth  14-19  20-29  30-39  40-49 | -6.58 (-8.11, -5.05)  2.72 (0.50, 1.04)  3.80 (2.38, 5.23)  -0.77 (-4.21, 2.68) | -21.18(-27.25,-14.94)  0.97 (-0.95, 2.89)  3.71 (0.36, 7.05)  1.49 (-9.4,12.39) | -11.52(-17.53,-5.42)  1.52 (-0.46, 3.49)  0.93 (-2.33, 4.18)  -0.60 (-9.90, 8.72) |
| Current marital status  Never married  Married/partner  No longer together | 16.52 (9.58, 23.30)  -0.55 (-0.75, -0.34)  7.44 (1.38, 13.44) | 2.22(-10.89, 15.26)  -0.02 (-0.53, 0.57)  -1.55 (-14.44,11.38) | 25.89 (9.98, 40.50)  -0.10 (-0.57,-0.33)  -14.64 (-24.91,-4.04) |
| Decision maker on spending of mothers income  Mother alone  mother& spouse  spouse alone  No income mother /missing | -2.27 (-3.29, -1.25)  15.50 (12.41, 18.56)  -0.28 (-3.33, 2.78)  -1.29 (-2.14, -0.44) | 0.69 (-1.87, 3.25)  9.02 (3.26, 14.73)  1.16 (-6.79, 9.10)  -4.40 (-7.32, -1.47) | 1.68 (-0.81, 4.16)  14.67 (8.42, 20.81)  -3.57 (-4.71, 11.81)  -7.02 (-9.69, -4.34) |
| Media exposure  No  Yes | -8.25 (-9.11, -7.39)  6.30 (5.64, 6.96) | -19.97(-24.99,-14.84)  2.81 (2.10, 3.53) | -19.50(-23.54,-15.39)  3.56 (2.82, 4.31) |
| Household wealth  Poor  Moderate  Rich | -2.57 (-3.36, -1.80)  2.22 (1.29, 3.15)  7.56 (3.46, 11.62) | -0.08 (-3.55, 3.40)  0.04 (-1.32, 2.18)  -2.72 (9.69, 4.29) | 0.08(-1.74, 1.90)  -0.16 (-2.85, 2.54)  0.85 (-15.72, 17.37) |
| COMMUNITY |  |  |  |
| Region  Northcentral  Northeast  Northwest  Southeast  Southsouth  Southwest | 5.83 (3.86, 7.81)  -6.00 (-7.25, -4.74)  -12.21 (-13.08,-11.35)  23.22 (19.12, 27.23)  20.15 (17.41, 22.86)  12.60 (8.95, 16.22) | -2.81 (-8.63, 3.02)  -23.25(-28.20,-18.18)  -22.08(-26.37,-17.70)  11.46 (7.02, 15.84)  19.69 (13.38, 25.85)  6.37 (2.95, 9.78) | 10.19 (5.31, 15.02)  -17.89(-21.21,-14.54)  -20.59(-24.05,-17.09)  11.67 (5.24, 18.00)  10.78 (4.62, 16.85)  21.05 (16.21, 25.80) |
| Distance to nearest Health Facility  No/Not a big problem  Big problem | 3.28 (2.62, 4.00)  -4.49 (-5.40, -3.58) | 1.25 (0.21, 2.29)  -5.52 (-10.10,-0.93) | 1.54 (0.65, 2.44)  -8.11 (-12.78,-3.40) |
|  |  |  |  |
